# Investigation into the antimicrobial action and mechanism of a novel endogenous peptide β-casein 197 from human milk

**DOI:** 10.1186/s13568-017-0409-y

**Published:** 2017-06-06

**Authors:** Yanrong Fu, Chenbo Ji, Xiaohui Chen, Xianwei Cui, Xing Wang, Jie Feng, Yun Li, Rui Qin, Xirong Guo

**Affiliations:** 1 Nanjing Maternal and Child Health Medical Institute, Nanjing Maternal and Child Health Hospital, Obstetrics and Gynecology Hospital Affiliated to Nanjing Medical University, 123 Tianfei Lane, Mochou Road, Nanjing, 210004 China; 2 Department of Child Health Care, Jiangsu Women and Children Health Hospital, Women and Child Branch Hospital of Jiangsu Province Hospital, The First Affiliated Hospital with Nanjing Medical University, Nanjing, 210036 China; 3 Shenzhen Easyhin Technology Co., Ltd, Shenzhen, 518000 China

**Keywords:** Human milk, β-Casein 197, Common pathogenic bacteria, Antibacterial effect

## Abstract

A novel endogenous peptide cleaved from 197–213 AA of β-casein, named β-casein 197, was identified by tandem mass spectrometry. β-casein 197 constituted a significant proportion of the peptide content in preterm milk. This study investigated the antibacterial effects and mechanisms against common pathogenic bacteria. Six bacterial strains were selected for this study: *Escherichia coli*, *Staphylococcus aureus*, *Yersinia enterocolitica*, *Listeria monocytogenes*, *Klebsiella pneumonia* and *Bacillus subtilis.* After synthesis, serial twofold dilutions of β-casein 197 were added to select for sensitive bacteria. The disk diffusion method and analysis of bacterial staining were used to identify antibacterial effect, while DNA-binding, scanning electron microscopy and transmission electron microscopy were used to explore antimicrobial mechanisms. Disk diffusion showed that *E. coli*, *S. aureus* and *Y. enterocolitica* were sensitive to the β-casein 197. In addition, live/dead fluorescent staining also confirmed antibacterial effects. Scanning electron and transmission electron microscopy revealed affected extracellular and intracellular structure for three species of bacteria, while a DNA-binding assay showed that the antimicrobial activity did not occur through DNA binding. This study suggests that β-casein 197 has antimicrobial activity against common pathogenic bacteria in newborns with infection. The peptide induced membrane permeabilization but did not bind to genomic DNA. Based on our findings, β-casein 197 has potential clinical value for preventing infections of premature infants.

## Introduction

As a result of biological evolution, breast milk provides perfect nutrition for newborns, containing a variable and complex composition. According to numerous studies, the composition of breast milk differs with regard to multiple factors, fitting requirements in accordance with each infant’s characteristics (Andreas et al. 2015; Ovali et al. 2006). Breast milk is the most beneficial food for all infants, whether preterm or full-term, and is produced specifically by each mother to satisfy her unique infant. Breastfeeding is not only the best way to provide newborns with abundant nutrients but also protects against infection such as sepsis, pneumonia and enteritis, particularly in premature infants (Gidrewicz and Fenton 2014). Breast milk reduces the load and translocation of gastrointestinal flora, reduces intestinal permeability, and lowers the incidence of infections (Baricelli et al. 2015; Schroeder et al. 2011; Sheng et al. 2014; Trend et al. 2015). Breast milk supports not only essential nutrients but also bioactive factors that have the ability to inhibit various diseases.

Endogenous peptides found in human breast milk are an area of increasing interest. It has previously been shown that breast milk contains hundreds of endogenous peptides derived from proteins by specific proteases, many of which had antibacterial action (Andreas et al. 2015; Khaldi et al. 2014). Endogenous peptides present in human milk were greater with preterm birth than full term (Dallas et al. 2015). Preterm milk is produced in immature mammary glands, which changes the synthesis and secretion processes of proteins, leading to differences in enzymatic activity that results in varied peptide profiles. However, more research is needed to elucidate the potential mechanisms of these endogenous peptides.

We recently used tandem mass spectrometry (MS/MS) to identify the peptides in both term and preterm human milk, and identified a peptide derived from β-casein: a sequence (197–213) from human β-casein. According to the naming method of a previous study (Plaisancie et al. 2015), we named this peptide β-casein 197. Using an online database, we discovered that the peptide, composed of 17 amino acid residues, was a stable endogenous peptide with a long half-life. Many researchers discovered that a multitude of endogenous peptides had antimicrobial activities. The mechanism of antimicrobial activity against bacteria was mostly via permeabilization of the membrane and DNA binding, which could explain the antimicrobial activity of human milk (Hakansson 2015). These peptides could be developed as potential ingredients of health-promoting foods supplied into formula, and even therapeutics (Mohanty et al. 2016). Here, we investigated the antibacterial effect and mechanism of β-casein 197 against common pathogenic bacteria in newborns with infection.

## Materials and methods

### Characteristics

We revealed the characteristics of β-casein 197 using the online database, Peptide Atlas (http://www.peptideatlas.org/). The peptide isoelectric point (pI) and molecular weight (Mw) were calculated by an online pI/Mw tool (http://web.expasy.org/compute_pi/). The ProtParam tool was used to review the physical and chemical parameters of β-casein 197, and the helical wheel was used to illustrate the alpha helices. The BindN tool (http://bioinfo.ggc.org/bindn/) was used for sequence-based prediction.

### Synthesis of peptides and bacterial strains

β-Casein 197 (LLNQELLLNPTHQIYPV) was synthesized by Science Peptide Biological Technology Co., Ltd. (Shanghai, China) using the solid-phase method. We used six bacterial strains in this study. *Escherichia coli* (*E. coli*, ATCC25922), *Staphylococcus aureus* (*S. aureus*, ATCC25923), and *Yersinia enterocolitica* (*Y. enterocolitica*, ATCC23715) were obtained from the American Type Culture Collection. *Listeria monocytogenes* (*L. monocytogenes*), *Klebsiella pneumonia* (*K. pneumonia*) and *Bacillus subtilis* (*B. subtilis*) were offered by Nanjing Normal University, China.

Thirty microliters of each culture was used to inoculate 3 mL of fresh Luria–Bertani (LB) (tryptone 10 g L^−1^, yeast extract 5 g L^−1^, NaCl 10 g L^−1^), and cultures were incubated at 37 °C with shaking until the bacteria reached the exponential phase of growth. A Millipore Scepter 2.0 (USA) was used to adjust the bacterial concentration to 5000–10,000 colony-forming units per milliliter (CFU mL^−1^).

### Antibacterial susceptibility assays

Serial twofold dilutions of β-casein 197 were added to the same number of log-phase bacteria in 96-well plates. The final concentrations of β-casein 197 were 25, 12.5, 6.25, 3.13, 1.56, 0.78 and 0 μg mL^−1^. Then, the plates were incubated at 37 °C for 24 h, and turbidity was measured at 630 nm using a Synergy HT multidetection microplate reader (Synergy HT, Bio-Tek Instruments, USA).

### Disk diffusion test

Yeast nitrogen broth agar plates were inoculated with bacterial suspensions in mid-logarithmic phase by swabbing the agar surface. After the plates were allowed to dry, sterile paper disks containing β-casein 197 (6.25 μg mL^−1^) and double-distilled H_2_O (ddH_2_O) were placed on the agar plate surface. The resulting zones of inhibition were measured after incubation at 37 °C for 24 h.

### Staining bacteria analysis

A solution of the LIVE/DEAD BacLight Kit L13152 (Thermo Fisher, USA) was prepared by dissolving the contents of component A and B in 5 mL of filter-sterilized ddH_2_O. The final concentration of each dye was 6 µM for the green-fluorescent nucleic acid stain (SYTO 9) and 30 µM for the red-fluorescent nucleic acid stain (propidium iodide, PI). The green stain labels bacteria with intact membranes, while the red stain penetrates damaged membranes.

We prepared 25 mL (~1 × 10^5^ CFU mL^−1^) of bacterial culture exposed to 25 μg mL^−1^ β-casein 197 or ddH_2_O by centrifugation at 100×*g* for 10 min followed by removal of the supernatant. Then, we re-suspended the pellet in 2 mL of 0.85% NaCl. After adding 1 mL of this suspension to a 45 mL centrifuge tube containing 20 mL of 0.85% NaCl, the tube was incubated at room temperature for 1 h with mixing every 15 min. The bacteria were collected by centrifugation at 100×*g* for 10 min, washed twice with 0.85% NaCl and re-suspended in 10 mL of 0.85% NaCl. The staining reagent mixture and the preparation of bacterial suspensions were mixed thoroughly and incubated at room temperature in the dark for 15 min. A volume of 5 µL of the stained bacterial suspension was trapped between a slide and an 18 mm square coverslip and was observed using a fluorescence microscope (Carl Zeiss, Observer D1).

### DNA binding assay

Gel-retardation experiments were performed using 5 µL of a 25 µg mL^−1^ pBR322 vector from *E. coli* (BioLabs, New England). Plasmid DNA (pDNA) was exposed to 5 µL of different concentrations of β-casein 197 at 37 °C for 1 h prior to gel electrophoresis of the reaction mixtures through a 0.7% agarose gel in Tris–acetate EDTA buffer. The gel was stained with Goodview™ (Sbsbio, China) and viewed with AlphaImager Mini System (Proteinsimple, USA).

### Scanning electron microscopy and transmission electron microscopy

*Escherichia coli*, *S. aureus* and *Y. enterocolitica* cells from exponential growth phase were concentrated after centrifugation at 13,000×*g* for 3 min, washed with 0.1 mol L^−1^ PBS and re-suspended in the same buffer. Millipore Scepter 2.0 (America) was used to adjust the cell density to 1 × 10^8^ CFU mL^−1^ and cells were exposed to a final concentration of 100 µL mL^−1^ β-casein 197 at 37 °C for 1 h. The cells were then centrifuged at 13,000×*g* for 3 min, washed with 0.1 mol L^−1^ PBS twice, and the supernatant was removed. Finally, the samples were prepared for scanning electron microscopy (SEM) and transmission electron microscopy (TEM) according to the previously published method (Taute et al. 2015).

### Statistical analysis

Statistical analysis was performed using the SPSS17.0 software package. Data in figures were presented as the mean ± standard deviation (SD). *P* < *0.05* were considered statistically significant.

## Results

### Characteristics of β-casein 197

**Fig. 1:**
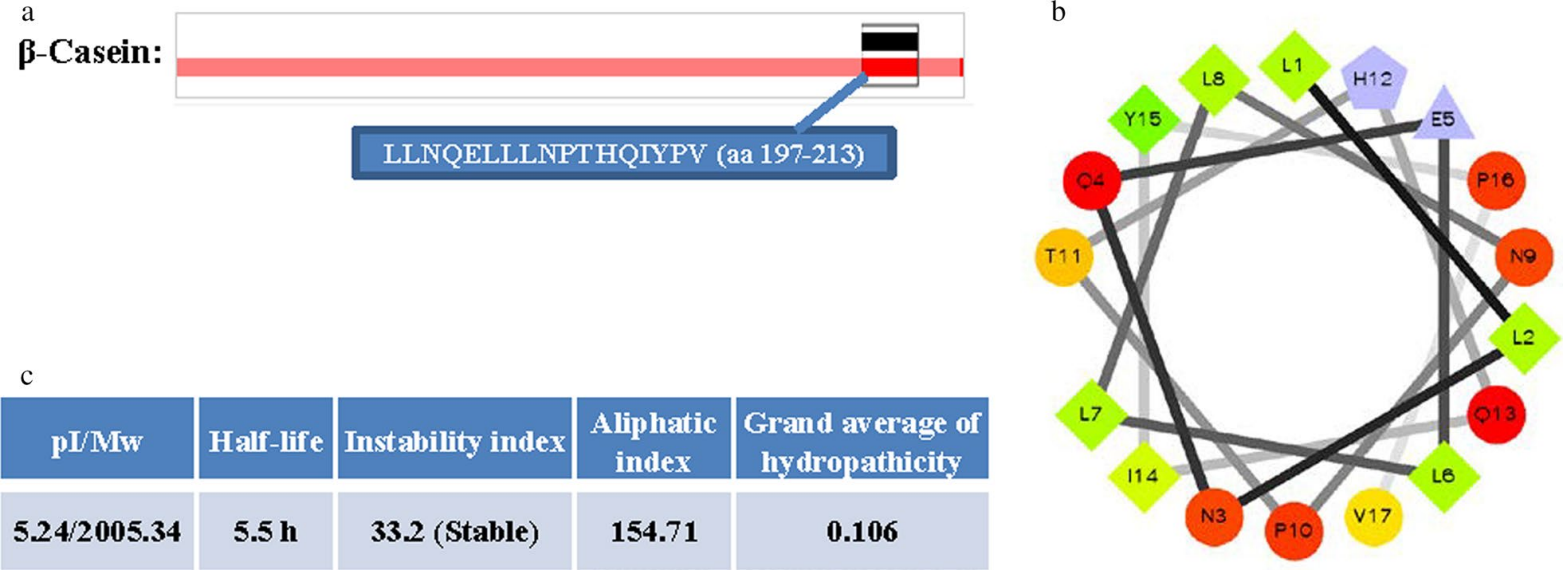
Characteristics of β-casein 197. **a** β-Casein 197 is a 17-amino-acid antimicrobial peptide that corresponds to amino acids 197–213 of β-casein. **b** The biochemical information of β-casein 197. **c** The helical wheel plot of β-casein 197. The output presents the hydrophilic residues as *circles*, hydrophobic residues as *diamonds*, potentially negatively charged residues as *triangles*, and potentially positively charged residues as *pentagons*. The most hydrophobic residue is *green*, and the amount of *green* decreases proportionally to the hydrophobicity, with zero hydrophobicity coded as *yellow*. Hydrophilic residues are coded *red* with *pure red* being the most hydrophilic residue, and the amount of *red* decreasing proportionally to the hydrophilicity

According to analysis of the Peptide Atlas database (http://www.peptideatlas.org/), β-casein 197 is a 17 amino acid antimicrobial peptide that corresponds to amino acids 197–213 of β-casein (Fig. 1a). According to the pI/Mw tool (http://web.expasy.org/compute_pi/), the pI and Mw of this peptide was 5.24 and 2005.34 Da, respectively (Fig. 1b). The low Mw of the peptide indicates that β-casein 197 cannot easily be hydrolyzed by protease. The instability was computed to be 33.2, which classifies the peptide as stable, meaning the peptide is unlikely to be destroyed by stomach acid. The aliphatic index and grand average of hydropathicity were 154.71 and 0.106. The helix-wheel plot of β-casein 197 revealed that the hydrophobic and hydrophilic amino acids were in alternating order, indicating that β-casein 197 may kill cells by means of being combined with membranes (Fig. 1c).

### Antimicrobial activity against bacteria

**Fig. 2:**
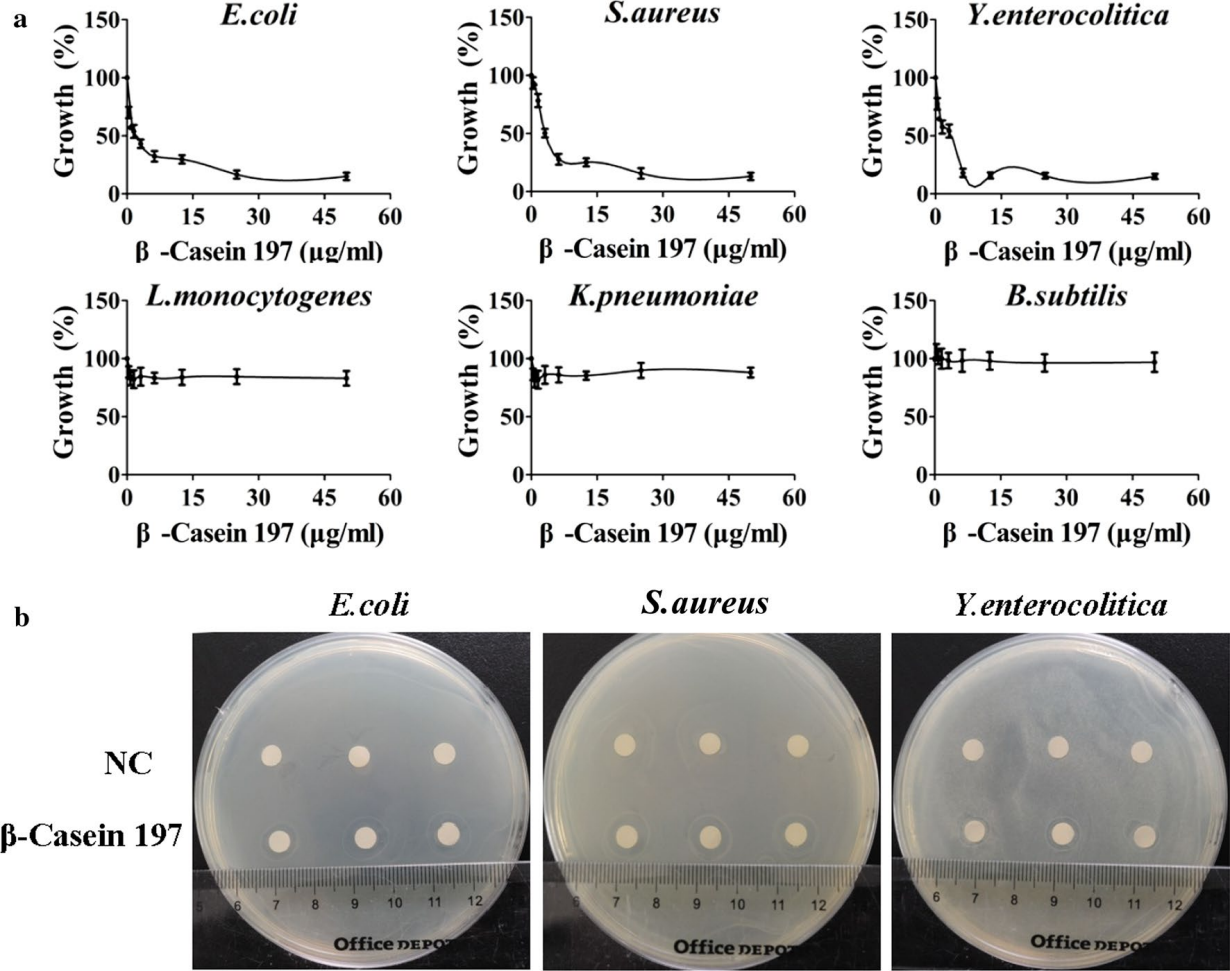
Antimicrobial activity of β-casein 197. **a** After incubation at 37 °C for 24 h, bacterial viability of *E. coli*, *S. aureus*, *Y. enterocolitica*, *L. monocytogenes*, *K. pneumonia* and *B. subtilis* after treatment with different concentrations of β-casein 197. **b** Inhibition zone diameters of *E. coli*, *S. aureus* and *Y. enterocolitica*. *NC* native control

We found only three bacterial strains, *E. coli*, *S. aureus* and *Y. enterocolitica* were significantly sensitive to β-casein 197 (Fig. 2a). The growth of these strains was inhibited nearly 50% after 24 h of culture. In contrast, *L. monocytogenes, K. pneumonia and B. subtilis* were not sensitive to β-casein 197 (Fig. 2a), and the inhibition rates were less than 20%. The disk diffusion method was used to additionally assess the susceptibility of *E. coli*, *S. aureus* and *Y. enterocolitica*. There were no zones of inhibition surrounding the disks that contained only ddH_2_O. In contrast, disks containing β-casein 197 exhibited zones of inhibition averaging 3 mm (Fig. 2b).

### Antimicrobial activity against bacteria via membrane permeabilization

**Fig. 3:**
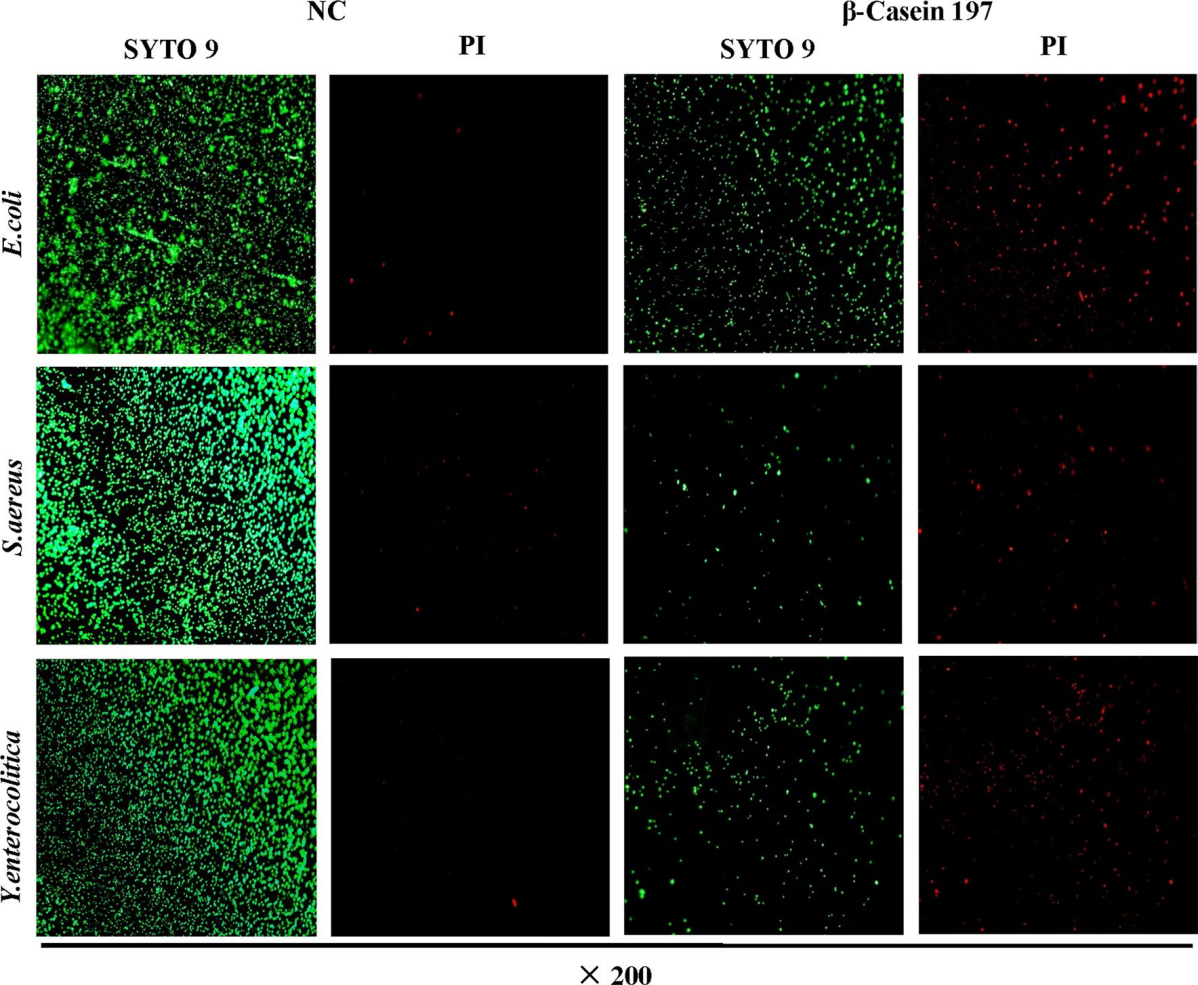
Microscopic assessment of *E. coli*, *S. aureus* and *Y. enterocolitica*, after treatment with β-casein 197. Fluorescence microscopy pictures of *E. coli*, *S. aureus* and *Y. enterocolitica*, control (*left two column figures*) and β-casein 197 treated (*right two column figures*). *Green cells* intact cells; *red cells* cells with a damaged membrane. *PI* propidium iodide, *NC* native control

Membrane damage was monitored using fluorescent dyes and fluorescence microscopy. β-Casein 197-treated bacteria exhibited decreasing green fluorescence and increasing red fluorescence (Fig. 3). Penetration of red dye into the damaged membranes led to a diminution of green fluorescence. *E. coli*, *S. aureus* and *Y. enterocolitica* displayed similar results.

**Fig. 4:**
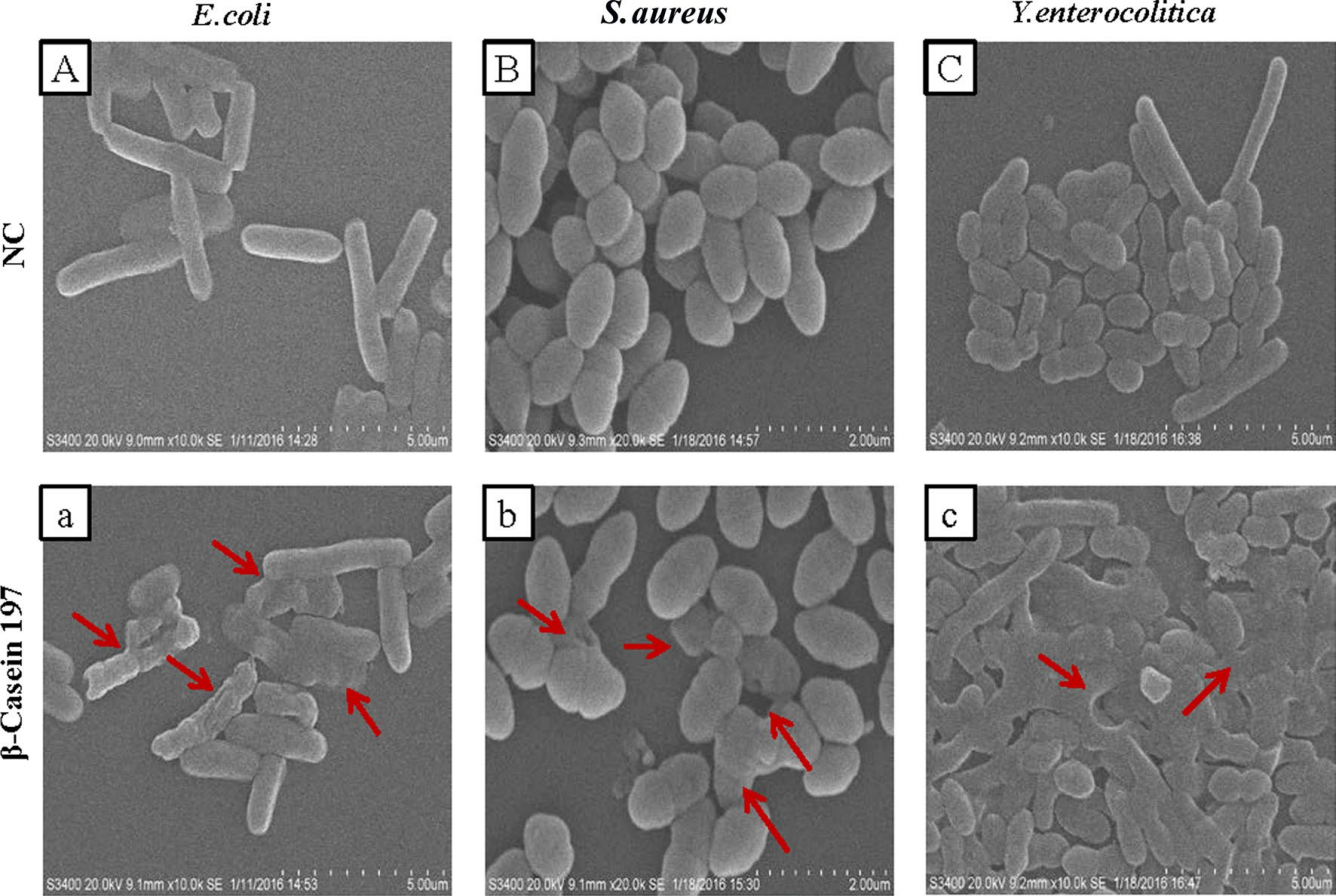
Morphology of *E. coli*, *S. aureus* and *Y. enterocolitica* cells were observed with scanning electron microscopy (SEM). **A**–**C** Control *E. coli*, *S. aureus* and *Y. enterocolitica* SEM images; **a**–**c** β-casein 197 treated samples. *NC* native control. *Arrows* indicate damage points

**Fig. 5:**
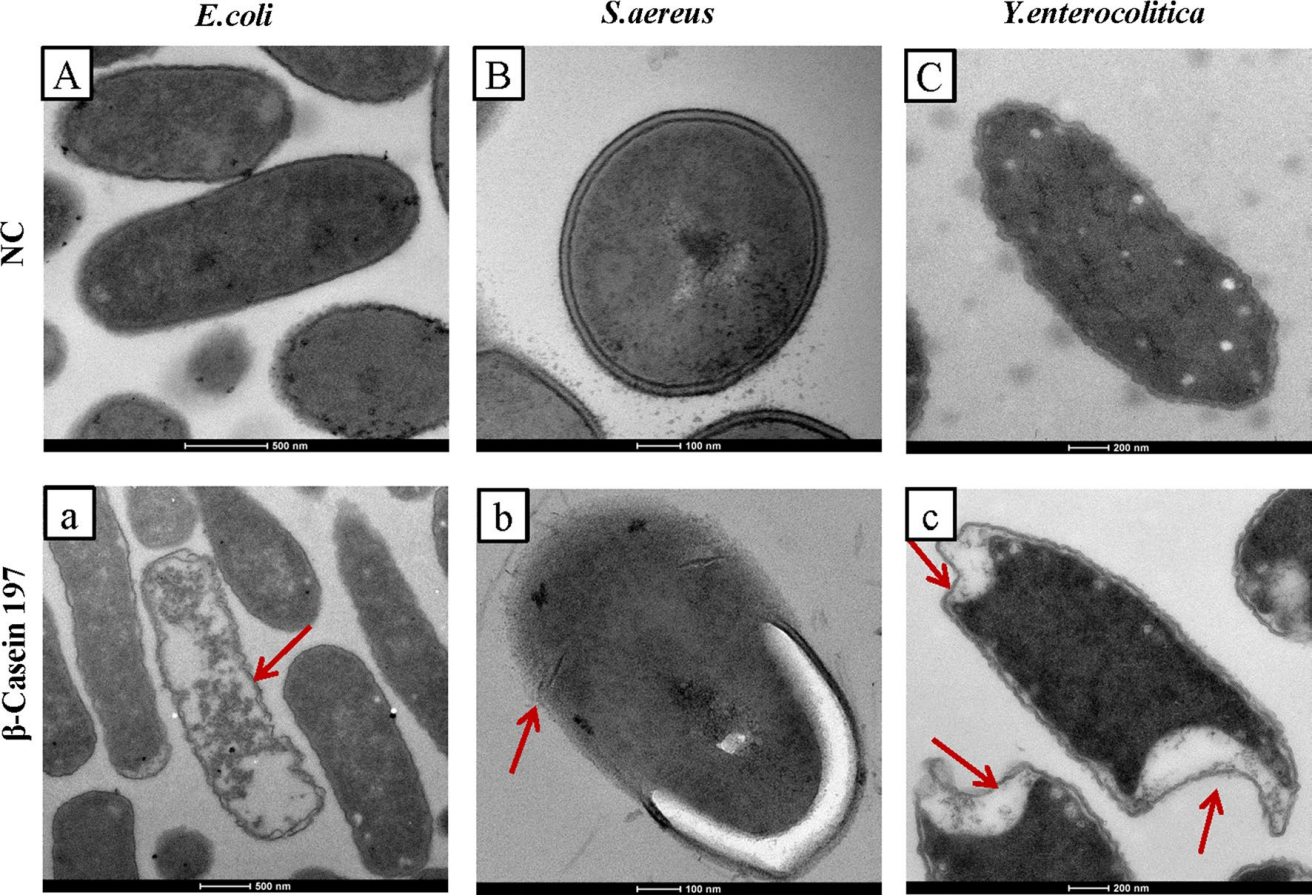
Morphosis of *E. coli*, *S. aureus* and *Y. enterocolitica* cells were observed under transmission electron microscopy (TEM). **A**–**C** Normal *E. coli*, *S. aureus* and *Y. enterocolitica* TEM images; **a**–**c** β-casein 197 treated samples. *NC* native control. *Arrows* indicate damage points

**Fig. 6:**
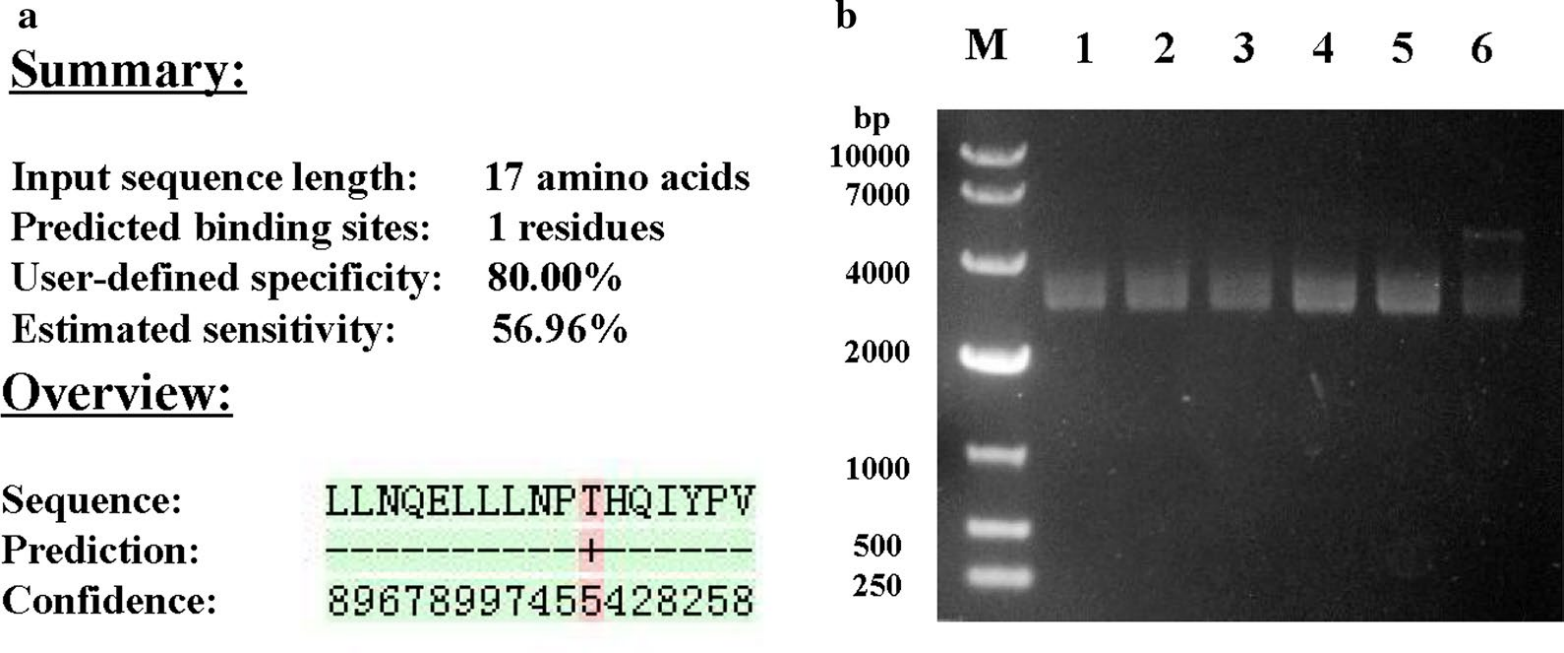
The DNA-binding properties of β-Casein 197. **a** ProtParam analysis of predicted binding residues of β-casein 197, predicted binding residues was labeled with a *red* “+”. **b** Gel retardation assays. Binding was assayed by the migration of pDNA. Various concentrations of peptides were incubated with 5 µL of 25 µg mL^−1^ pBR322 vector from *E. coli* at 37 °C for 1 h, and then the reaction mixtures were applied to 0.7% agarose gel electrophoresis. *Lane M* DNA marker DL 10,000; *Lane 1* 5 µL pDNA as control; *Lanes 2–6* mixture of pDNA and various concentrations of peptides (12.5, 25, 50, 100 and 500 µg mL^−1^)

In the pictures from SEM, complete and continuous membrane structures were observed distinctly in untreated bacteria (Fig. 4A–C), while treated bacteria showed disorganized membrane structures. Bacterias exposed to β-casein 197 had a wrinkled surface compared to the smooth surface of untreated bacteria (Fig. 4a–c). The damaged membranes of *E. coli*, *S. aureus* and *Y. enterocolitica* were also clearly identified in the pictures from TEM (Fig. 5). Compared with normal bacterial controls (Fig. 5A–C), numerous bleb-like structures, membrane ruffling, cytoplasmic retraction, intracellular clumping and indentation of the cell envelope were observed with TEM in β-casein 197 exposed group (Fig. 5a–c). The bindN tool for sequence-based prediction revealed that β-casein 197 had only one DNA- and RNA-binding residue (Fig. 6a). To determine whether β-casein 197 had DNA-binding capabilities, we performed a gel retardation assay. According to previous studies (Bandyopadhyay et al. 2013), the migration of pDNA was retarded as the quantity of the peptide, Lasioglossin II, increased due to binding of pDNA. However, we observed that the migration of different weight ratios of β-Casein 197 was the same as untreated samples (Fig. 6b).

## Discussion

Breast milk is recommended feeding for all newborns including premature infants (Boland 2005; Section on 2012). Breast milk is essential for the promotion of growth and development and vital in protecting infants from infections. In this article, we discovered a new endogenous peptide derived from β-casein named β-casein 197. We found that this peptide had antimicrobial effects on common bacterial pathogens such as *E. coli*, *S. aureus* and *Y. enterocolitica.* The antimicrobial mechanism of action of β-casein 197 was found to be membrane interaction instead of DNA-binding. We predicted that β-casein 197 might play an important role in the function of human milk against infection.

The formation of a mature immune system and establishment of the microbiota is vital for protecting infants from infections in an environment that is filled with harmful micro-organisms. Based on previous research, the current hypothesis is that certain innate immune cells compensate for the newborn’s impaired immune system and play an essential role against microbes during the initial phase of life (Kai-Larsen et al. 2014). Numerous studies noted that breastfed infants were better protected against infections than those fed with formula (Lamberti et al. 2013; Spatz and Edwards 2016). This observation is most likely due to multiple factors in breast milk that confer defense against pathogenic microbes throughout the intestinal mucosa. For the past 30 years, scientists observed that high levels of secretory immunoglobulin A and oligosaccharides could prevent adherence of harmful microbes to the gut mucosa (Gibbs et al. 1977; Prentice et al. 1985). Currently, increasing numbers of studies seek to improve understanding of the importance of breast milk, especially antimicrobial components such as antimicrobial peptides (AMPs), against infections (Hakansson 2015; Lepage and Van de Perre 2012; Ovali et al. 2006). Researchers hope to improve the biological activity of formula milk.

Peptidomics is considered to be a systematic, quantitative and comprehensive analysis of the small fraction of proteins present in breast milk samples (Guerrero et al. 2014). Endogenous peptides are produced from their corresponding proteins through specific proteases present in the identical biological system. A previous study revealed almost 700 endogenous peptides from 30 different proteins in human milk through extensive mass spectrometry analysis (Guerrero et al. 2014). However, 95% of the identified peptides were proteolytic products from the following four proteins: β-casein, α_s1_-casein, osteopontin and polymeric immunoglobulin receptor. As both β-casein and α_s1_-casein are highly abundant proteins in breast milk, they were expected to contribute the most to the peptide products. Because of their loose structures, many cleavage sites in these peptides are exposed to proteolytic enzymes. Meanwhile, studies found that low-molecular-weight casein fragments were more abundant in preterm milk compared with term milk (Dallas et al. 2015).

As we know, neonatal infections such as Late-onset neonatal sepsis, necrotizing enterocolitis (NEC), and pneumonia are usually caused by co-infection of bacterial strains including *E. coli*, *S. aureus* and *Y. enterocolitica*. *E. coli* is one of the most-studied microorganisms worldwide (Vila et al. 2016). Neonatal sepsis caused by *E. coli* represents a substantial worldwide public health problem. An important contributing factor to the problem is the increase of antimicrobial resistance by bacteria such as resistance to ampicillin. In the WHO report, within a wide range of infectious agents, including *E. coli*, *S. aureus* and *Y. enterocolitica,* antimicrobial resistance has reached worrisome levels that limit the development of modern medicine. Fortunately, breast milk contains secretary immunoglobulin A, oligosaccharides and AMPs, and is the best way to provide newborns with abundant nutrients and protection against infections.

AMPs destroy bacteria in a unique way that does is less prone to resistance. However, although hundreds of AMPs have been isolated, only a few have been investigated for their possible mode of action. The generally accepted mode of action for AMPs against Gram-negative bacteria is that the AMPs interact with the highly negatively charged surface of the membrane consisting of lipopolysaccharide. Then, the AMPs insert and translocate to the outer bilayer to bind the anionic inner membrane by a self-promoted uptake pathway (Hancock 2001; Mangoni et al. 2004). However, some studies discovered that rapid killing occurs due to serious reduction of membrane integrity at high concentrations. Membrane activity for the AMPs Os and Os-C may have intracellular targets such as DNA, similar to melittin at low concentrations (Taute et al. 2015). The killing mode of AMPs is complex and remains incompletely understood.

The present study addressed whether and to what extent this new peptide affects membrane permeation or combines with bacterial DNA and how it relates to cell viability and morphological changes of treated bacteria. According to the analysis of β-casein 197, the peptide is a stable 17-amino-acid antimicrobial peptide hydrolyzed from β-casein. A helix-wheel plot revealed that its antimicrobial activity may act through combining to membranes. Through these characteristics, β-casein 197 can diffuse through negatively charged cell walls of bacteria similarly to other peptides such as snakins and human β-defensins (Baricelli et al. 2015; Herbel and Wink 2016; Sheng et al. 2014). We discovered that *E. coli*, *S. aureus* and *Y. enterocolitica* were more susceptible to β-casein 197. Fluorescent dye was used to identify the action of AMPs against bacteria (Diels et al. 2005), and we easily observed membrane damage through fluorescence microscopy. As expected, the proliferations of three types of bacteria were all inhibited. The reduction of green fluorescence light as well as the increase of red light was observed as an indication that β-casein 197 treatment caused bacterial membrane damage, and this damage might be one of causes for the death of viable bacteria.

The unique effect of β-casein 197 on *E. coli*, *S. aureus* and *Y. enterocolitica* was further demonstrated by SEM and TEM, which showed wrinkled surfaces, bleb-like structures, destroyed membranes and leaked cytoplasm compared with normal morphology. Furthermore, a DNA retardation assay did not support a different mode of killing action in different concentrations of β-casein 197 as was reported for AMPs such as human β-defensins and lasioglossins, which have the ability to bind DNA (Bandyopadhyay et al. 2013). However, we can’t exclude the possibility that β-casein 197 has other intracellular targets for bacterial killing such as stimulation of autolytic enzymes or inhibition of proteins essential to the bacterial life cycle.

Many unknowns remain, and investigating the composition of human milk, especially under different circumstances, can be challenging. Since AMPs are the product of evolution, this study provides insights into the content and function of human milk. Future studies should examine the role of β-casein 197 in animal models. We believe that these data could provide valuable information to improve the biological activity of formula milk and lead to development of new therapeutic approaches for protecting infants from neonatal infections.

## Abbreviations

MS/MS: tandem mass spectrometry
pI: isoelectric point
Mw: molecular weight
*E. coli*: *Escherichia coli*
*S. aureus*: *Staphylococcus aureus*
*Y. enterocolitica*: *Yersinia enterocolitica*
*L. monocytogenes*: *Listeria monocytogenes*
*K. pneumonia*: *Klebsiella pneumonia*
*B. subtilis*: *Bacillus subtilis*
LB: Luria–Bertani
CFU mL^−1^: colony-forming units per milliliter
ddH_2_O: double-distilled H_2_O
PI: propidium iodide
pDNA: plasmid DNA
SEM: scanning electron microscopy
TEM: transmission electron microscopy
SD: standard deviation
AMP: antimicrobial peptide
NEC: necrotizing enterocolitis
